# Tandem Mass Tagging Based Identification of Proteome Signatures for Reductive Stress Cardiomyopathy

**DOI:** 10.3389/fcvm.2022.848045

**Published:** 2022-06-13

**Authors:** Sini Sunny, Arun Jyothidasan, Cynthia L. David, Krishna Parsawar, Arul Veerappan, Dean P. Jones, Steven Pogwizd, Namakkal S. Rajasekaran

**Affiliations:** ^1^Division of Molecular and Cellular Pathology, University of Alabama at Birmingham, Birmingham, AL, United States; ^2^Analytical and Biological Mass Spectrometry Core Facility, The University of Arizona, Tuscon, AZ, United States; ^3^Division of Pulmonary, Critical Care and Sleep Medicine, Department of Medicine, New York University School of Medicine, New York, NY, United States; ^4^Department of Environmental Medicine, New York University School of Medicine, New York, NY, United States; ^5^Division of Pulmonary, Allergy, Critical Care and Sleep Medicine, Emory University, Atlanta, GA, United States; ^6^Comprehensive Cardiovascular Center, Department of Medicine, University of Alabama at Birmingham, Birmingham, AL, United States; ^7^Division of Cardiovascular Medicine, Department of Medicine, The University of Utah, Salt Lake City, UT, United States; ^8^Center for Free Radical Biology, University of Alabama at Birmingham, Birmingham, AL, United States

**Keywords:** reductive stress, caNrf2, myocardial proteome, Tandem Mass Tag proteomic analysis, speckle tracking echocardiography

## Abstract

Nuclear factor erythroid 2-related factor 2 (NRF2), a redox sensor, is vital for cellular redox homeostasis. We reported that transgenic mice expressing constitutively active Nrf2 (CaNrf2-TG) exhibit reductive stress (RS). In this study, we identified novel protein signature for RS-induced cardiomyopathy using Tandem Mass Tag (TMT) proteomic analysis in heart tissues of TG (CaNrf2-TG) mice at 6–7 months of age. A total of 1,105 proteins were extracted from 22,544 spectra. About 560 proteins were differentially expressed in TG vs. NTg hearts, indicating a global impact of RS on the myocardial proteome. Over 32 proteins were significantly altered in response to RS -20 were upregulated and 12 were downregulated in the hearts of TG vs. NTg mice, suggesting that these proteins could be putative signatures of RS. Scaffold analysis revealed a clear distinction between TG vs. NTg hearts. The majority of the differentially expressed proteins (DEPs) that were significantly altered in RS mice were found to be involved in stress related pathways such as antioxidants, NADPH, protein quality control, etc. Interestingly, proteins that were involved in mitochondrial respiration, lipophagy and cardiac rhythm were dramatically decreased in TG hearts. Of note, we identified the glutathione family of proteins as the significantly changed subset of the proteome in TG heart. Surprisingly, our comparative analysis of NGS based transcriptome and TMT-proteome indicated that ~50% of the altered proteins in TG myocardium was found to be negatively correlated with their transcript levels. In association with the altered proteome the TG mice displayed pathological cardiac remodeling.

## Highlights

- Redox scoring in Ca-Nrf2-TG mouse hearts revealed a reductive stress (RS) phenotype.
- Tandem mass tagging (TMT) proteomics identified about 560 differentially expressed proteins in RS mouse hearts.
- Post-translational modifications such as oxidation, N-ethylmaleimide, methionine loss and acetylation were observed in RS hearts.
- Disproportionate transcription vs. translation was evident in ~50% of DEPs in RS
- RS mediated myocardial dyssynchronicity is coupled with significantly altered proteome and transcriptome profiles.

## Introduction

A real-time action of Nrf2 in response to stress is vital to preserve the redox homeostasis in cells and tissues, but its activation under unstressed settings could tilt the redox balance toward the reductive arm, leading to reductive stress (RS). Cardiac-specific transgenic mouse models expressing constitutively active Nrf2 (caNrf2-TG) develop RS and cause pathological cardiac remodeling (1). We recently reported that caNrf2-TG mice exhibit eccentric cardiac hypertrophy with increased ejection fraction and progressive diastolic dysfunction (1). Many studies from other investigators and we revealed global changes in the transcriptome and pathophysiological processes in the heart, skeletal muscle, metabolism, cancer, and neurodegeneration under reductive and hyper-reductive conditions (2–7). Likewise, caNrf2-TG mice displayed a unique transcriptome profile that is believed to drive the pathological remodeling of the myocardium in a chronic setting (8, 9). Nonetheless, the proteome alterations associated with RS and subsequent cardiac remodeling are unknown.

From the past decade, other studies indicated that a reductive intracellular environment (i.e., RS) might be challenging to normal physiological signaling processes due to a lack of basal reactive oxygen and nitrogen species (ROS/RNS) (10–15). Particularly, ROS/RNS are necessary for cell proliferation and differentiation during embryonic development, regeneration of stem cells/tissues and healing of damaged tissues, etc. (16–20). We recently reported that either acute or chronic reductive stress conditions impairs the regeneration of myoblasts and neuroblastoma cells (2, 3). Reductive stress is defined as the abnormal increase (≥2.0 fold) of reducing equivalents (e.g., GSH:GSSG; NADPH:NADP; Cysteine:Cystine, etc.) in a given cell or cellular compartment, which influences the basal reactive oxygen species (ROS) signaling mechanisms through shifting the redox (oxidant and reductant/antioxidant) toward the reductive arm. Notably, RS-induced neurodegeneration is driven by misfolding and aggregation of proteins (3), but the mechanisms are unknown.

In the present study, we tested the hypothesis that Nrf2 mediated chronic RS will alter the myocardial redox proteome and perturb the pathophysiological processes. The study reveals that cRS significantly alters the myocardial proteome in association with structural and functional remodeling.

## Materials and Methods

Heart-specific constitutively active Nrf2 (CaNrf2) transgenic (TG) and non-transgenic (NTg) mice (*n* = 4/group) at 6 months of age were used for analyzing the myocardial proteome using Tandem Mass Tagging (TMT) based mass-spectrometry. Detailed methods for animal maintenance, redox score, TMT proteomics, LC-MS/MS mass-spec, protein identification, Scaffold based-conformational changes, pathway analysis, bioinformatics, and speckle tracking echocardiography (STE) are provided in the Supplemental Section.

## Results

### Identification of Proteins in RS (TG) Hearts

**Figure 1:**
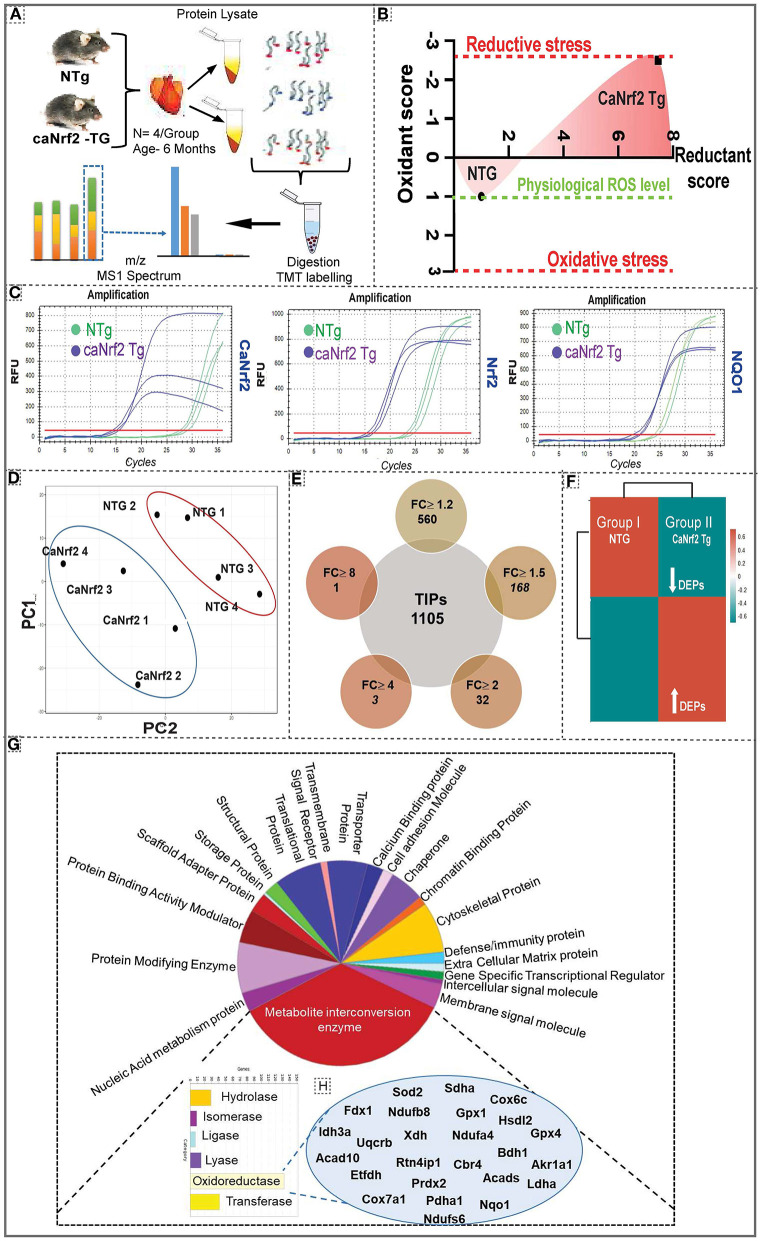
Effect of chronic reductive stress on myocardial proteome. **(A)** Overall methodology adopted for Tandem Mass Tagged LC-MS/MS analysis. Heart tissues were harvested from non-transgenic (NTg) and caNrf2-Tg (*N* = 4 mice/group) and protein concentration was determined in homogenates with BCA kit (Bio-Rad, USA). After trypsin digestion, peptides were reconstituted in 0.5 M TEAB and processed for TMT tagging (Tandem Mass Tag kit, ThermoFisher Scientific). LC-MS/MS analysis was performed on a Q Exactive Plus mass spectrometer (Thermo Fisher Scientific) equipped with an EASY-Spray nanoESI source. MS and MS/MS data were searched against the amino acid sequence of the Uniprot mouse protein database using Thermo Proteome Discoverer v 2.4.0.305 (Thermo Fisher Scientific). The protein and peptide identification results were further analyzed with Scaffold Q + S v 4.11.1 (Proteome Software Inc.). **(B)** Comprehensive approach used to calculate the “redox score” in TG by comparing to basal redox factors in NTg. Here, we included the levels of small molecular antioxidants (i.e. GSH, Cysteine/Cystine ratio); antioxidant proteins (i.e. GST MU, NQO1, CAT, GPX 1, SOD1, GCLM, GCLC, SOD2); antioxidant enzymatic levels (i.e. TAC); antioxidant gene expression (i.e. GCLM, NQO1, GSR, GST alpha, GCLC, GCLM, GSR, NQO1, GST MU, GPX 1, CAT) as well as the reactive oxygen species levels (i.e. DHE). **(C)** Quantitative genotyping in caNrf2 mice caNrf2 genotyping primer, endogenous primers for Nrf2, NQO1 and GCLC. **(D)** Principal Component Analysis (PCA) Plot generated using Total Identified Proteins (TIPs) showed segregation of NTg and CaNrf2 TG as distinct groups. **(E)** Venn diagram showing the number of Differentially Expressed Proteins (DEPs) based on different fold change (CaNrf2 TG vs. NTg) identified in TMT proteome software. **(F)** Global heat map generated using R studio for TIPs (1,105) proteins identified in TMT analysis. **(G)** Gene ontology pathway by PANTHER analysis. Protein Analysis THrough Evolutionary Relationships (PANTHER) analysis (http://pantherdb.org/) for biological function distributed the total identified proteins (TIPs) into different metabolic categories. About 25% of the proteome function grouped under metabolite interconversion category, with oxidoreductase enzyme family as the top upregulated one. The proteins identified under oxidoreductase group are shown in **(H)**.

Quantitative proteomic analysis was performed on NTg and TG heart tissues using six plex-tandem mass tag (TMT) labeling (Figure 1A; Supplementary Figure 1). Our comprehensive redox analysis confirmed the reductive stress in caNrf2-TG hearts (Figure 1B). Quantitative genotyping indicated the transgene expression (Ct values) in TG mice when compared to NTg, which did not amplify a PCR product for the primers that recognize only the truncated transgene (caNrf2) (Figure 1C). Of note, qPCR using a primer that recognizes endogenous mouse Nrf2, but not caNrf2, also showed a lower Ct value, suggesting auto-up-regulation of Nrf2 mRNA. Ct values for NQO1 (Nrf2 target) in TG hearts reveals the robustness of RS in these hearts. Principal compound analysis using 2D scatter plot (PCA plot) revealed distinct clustering of TG (caNrf2-TG) and NTg samples (Figure 1D), suggesting a clear impact for RS on myocardial proteome. At 95% protein threshold confidence level, a significant difference between proteome distributions in each group was observed. A total of 22,544 quantitated spectra (out of 24,372 threshold spectra) with 95% minimal False Discovery Rate (FDR) representing 1,105 proteins (889 clusters) were identified in the TG heart tissues. Comparative spectral profiling based on log fold change identified 560 differentially expressed proteins (DEPs) with a significant log_2_ fold change (FC) ≥ 1.2, 168 DEPs at FC ≥ 1.5, 32 DEPs at FC ≥ 2, 3 DEPs at FC ≥ 4 and 1 DEP at FC ≥ 8 in TG *vs*. NTg mouse hearts (*P* > 0.05) (Figure 1E). R studio analysis distributed the whole DEPs into distinct bins based on log_2_ fold change showing up/down regulated proteins in TG vs. NTg (Figure 1F).

### Gene Ontology and Pathway Analysis

To gain further insight into the enriched pathways associated with the RS, core proteome was submitted for Panther (Figure 1G) and String analysis (Supplementary Figure 2). Functional classification using Panther showed an enrichment of metabolite interconversion enzyme family (i.e., oxidoreductase family), which are directly or indirectly contributing to myocardial health, in TG hearts. Among the metabolic enzymes, oxidoreductases were 80% enriched in CaNrf2-TG vs. NTg hearts. Mainly, the protein targets of Nrf2, NQO1, GPX1, GPX4, SOD2 were detected by Panther in CaNrf2-TG hearts validates the signature for RS. Other proteins that are directly or indirectly associated with RS proteome are listed in Figure 1H. Furthermore, string analysis clustered the proteome core into different groups, based on the physical protein association, with NADPH as the most enriched network. Based on functional/physical protein associations and kmeans clustering method, string segregated the proteins into three clusters, which are tightly enriched and regulated by ubiquitin, GSR and stress protein families (Supplementary Figure 2).

### Top Enriched Proteins and Peptide Modifications in the Reductive Stress Hearts

**Figure 2:**
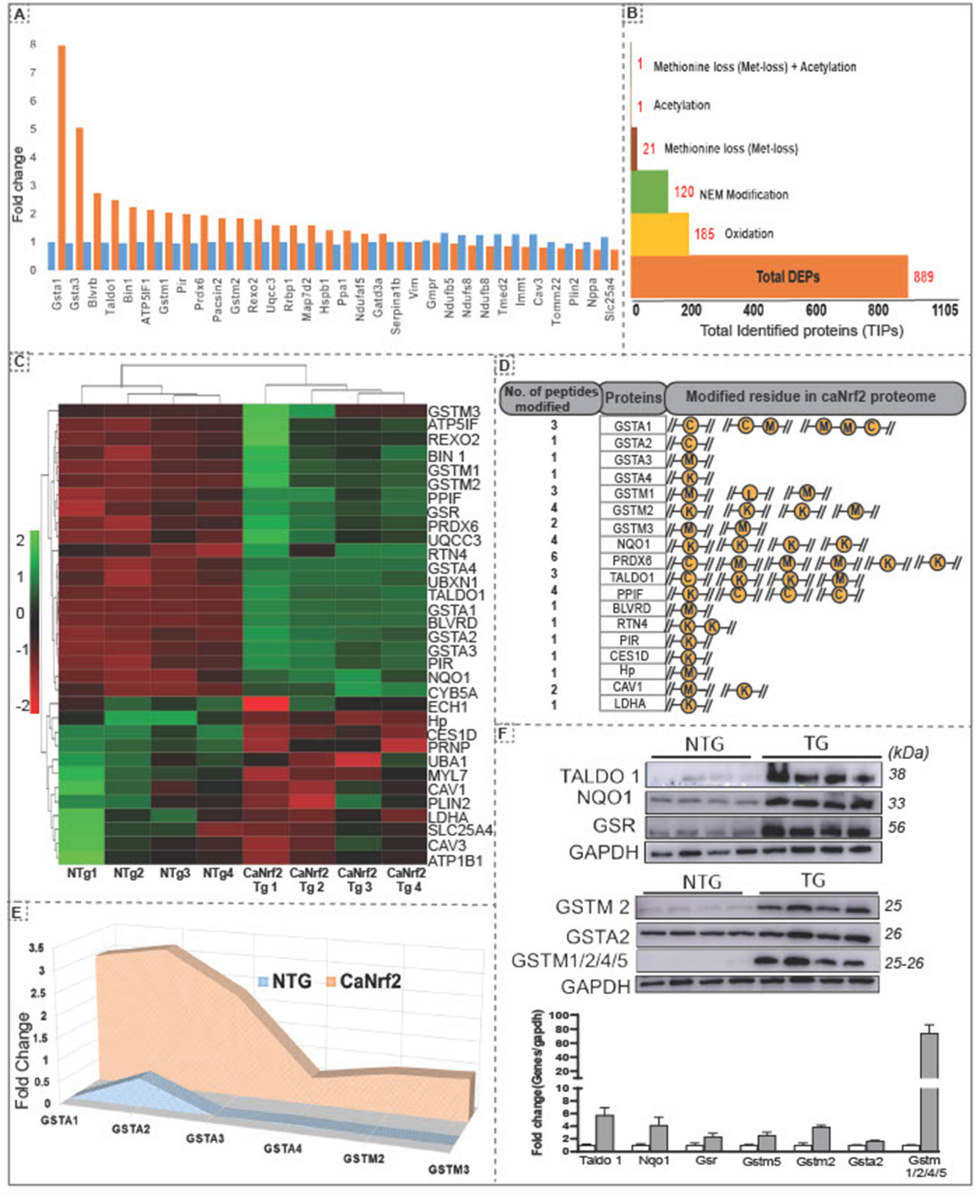
Hierarchical clustering of differentially expressed proteins and amino acid modifications in proteins in the RS hearts. **(A)** Unsupervised clustering of total identified proteins showed 32 DEPs at 2 fold change with Gsta1, Gsta2, Blvrd, Taldo1, Bin1, Atp5lf1, gstm1,Pir and Prdx6 as the top upregulated proteins in CaNrf2 hearts. **(B)** Spectrum view by proteome scaffold software identified different types of modification in differentially expressed proteins (889) with 185 proteins having oxidation,120 with NEM modification, 21 with loss of methionine peptide, 1 with acetylation, 1 with acetylation and 1 peptide with both loss of methylation and acetylation. **(C)** CaNrf2 proteome heat map using R studio for highly upregulated proteins (based on log2 FC) identified in scaffold software. **(D)** Highly upregulated proteins are associated with amino acid modifications in peptide(s). Data showing the number of peptides modified in each protein and modified residue in each peptide. A representative peptide is shown in yellow color. **(E)** TMT proteome identified GSR family of proteins as top enriched in CaNrf2 hearts in comparison with NTg. Significantly changed proteins are Gsta2, Gsta3, Gsta4, Gstm2, Gstm3 and Gsta1 (based on log2 fold change ratio). **(F)** Immunoblot validation for the selected proteins in TG vs. NTg hearts. Corresponding densitometry analysis using Image-J is shown.

Using unsupervised clustering, we identified 32 DEPs (FC ≥ 2) including GSTA1 and GSTA3 showing a highest enrichment in TG hearts (Figure 1E). Other proteins like PRDX6, TALDO1, BLVRD, BIN1, GSTM1 and PIR were recognized as the second big enrichment (Figure 2A). Of note, we identified several post-translational modifications such as oxidation (185), N-ethylmaleimide (NEM; 120), methionine loss (20), acetylation (1) and methionine loss + acetylation (1) in comparison with NTg (Figure 2B).

### Putative Indicators of Reductive Stress

Interestingly, among the DEPs (up- or down-regulated), we noticed some of them were intact while others had modifications at specific amino acid residues in the RS hearts (Figure 2C). A list of top identified proteins with their modifications in peptides and the amino acid residues is shown Figure 2D. Several proteins had modifications in cysteine, methionine, and lysine residues in CaNrf2-TG hearts. Glutathione metabolism is tightly regulated and has been implicated in myocardial redox signaling (21). Interestingly, we observed glutathione enzyme family and other related proteins were robustly upregulated in TG (FC > 2.7) in CaNrf2-TG vs. NTg (Figure 2E). Spectrum analysis of GSTA1, GSTA2, GSTA3, GSTA4, GSTM1, GSTM3 and GSTM4 recognized cysteine, lysine, and methionine modifications in their peptide fragments. In fact, we validated and confirmed the expression of some of these proteins using immunoblotting and quantitated their levels using Image-J (Figure 2F).

### Comparative Analysis of Transcription (NGS-RNA-Seq) and Translation (TMT-Proteomics) of Protein Targets in the RS Myocardium

**Figure 3:**
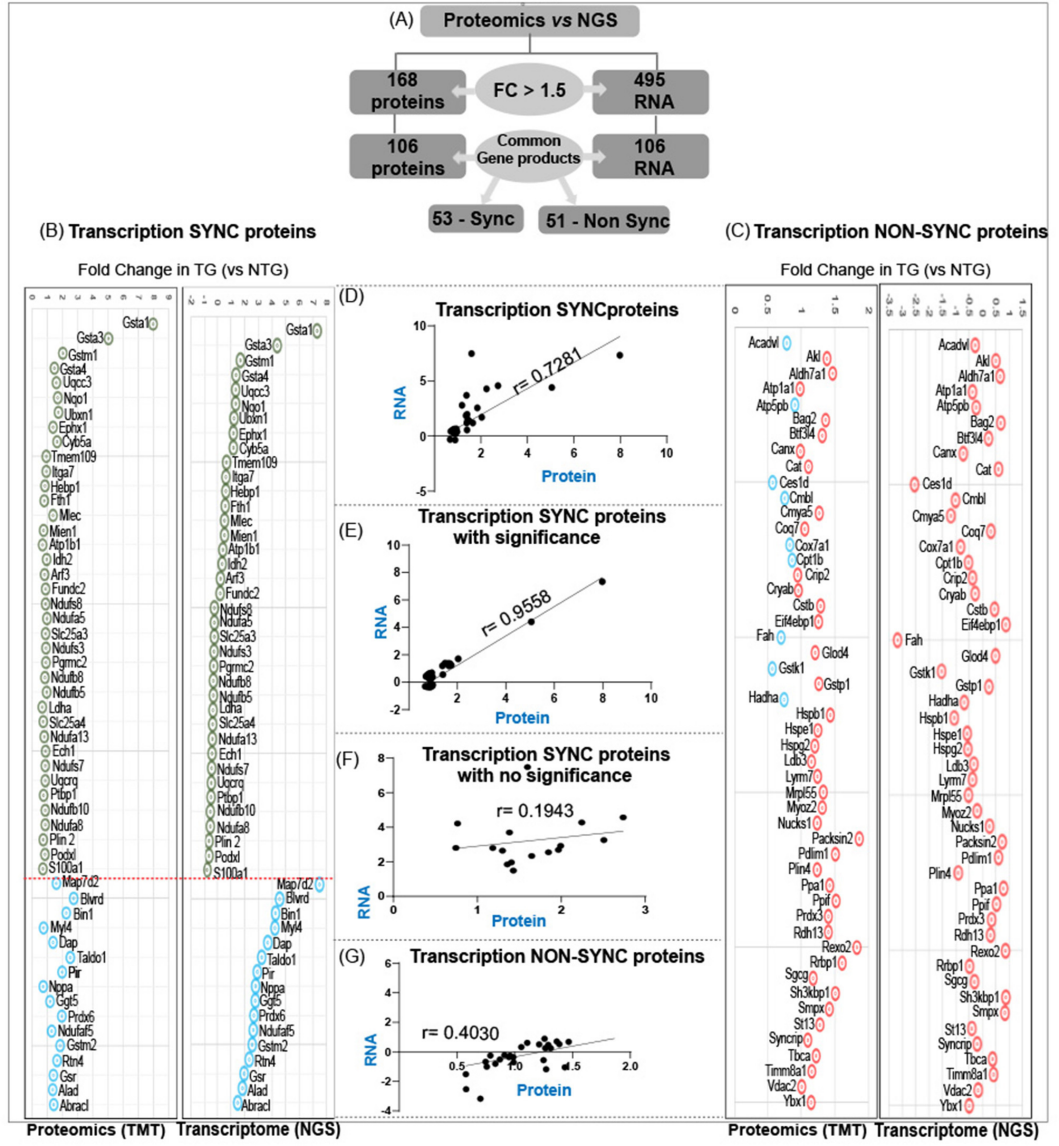
Analysis of transcription-sync versus transcription-non sync proteins in RS hearts. **(A)** Out of 106 commonly found gene products in NGS (RNA) and respective translational products (proteins by TMT) at a FC > 1.5, the levels of 50 proteins are not consistent with their RNA levels (either up or down regulated). Proteins that are steadily expressed according to their respective mRNA levels are classified as “transcription-sync proteins” **(B)** and the ones that do not match with their mRNA levels are termed as “transcription-nonsync proteins” **(C)**. **(D,E)** A strong positive correlation (*r* = 0.955) was observed among 38 of transcription-sync proteins **(C)**, but a weak positive correlation (*r* = 0.194) for 16 transcription-sync proteins **(F)**. Transcription-nonsync proteins showed a negative correlation **(G)**.

Comparison of RNA sequencing data [Next Generation Sequencing (NGS)] with proteomic data (TMT) from CaNrf2-TG hearts showed surprising results. Out of 104 commonly found gene products in NGS (RNA) and respective translational products (proteins by TMT) at a FC > 1.5, the levels of 50 proteins are not consistent with their RNA levels (either up or down regulated) (Figure 3A). The proteins that are steadily expressed according to their respective mRNA levels are classified as “transcription-sync proteins (T-SP)” (Figure 3B) and the ones that do not match with their mRNA levels are termed as “transcription-nonsync proteins (T-NSP)” (Figure 3C). Among the transcription-sync proteins, 16 (MAP7D2, BLVRD, BIN1, MYL4, DAP, TALDO1, PIR, NPPA, GGT5, PRDX6, NDUFAF5, GSTM2, RTN4, GSR, ALD, and BRACL) with marginally higher RNA levels were statistically insignificant (*r* = 0.194). As expected, changes in Nrf2 targeted antioxidant proteins are statistically significant (NQO1, GSR, GSTA1, GSTA3, GSTA4, and GSTM1) and grouped under “transcription-sync proteins.” Further, 9 of the “transcription-nonsync proteins” (ACADVL, ATP5PB, CES1D, CMBL, COX7A1, CPT1B, FAH, GSTK1, and HADHA) showed high protein levels albeit their significantly down regulated mRNA expression (Figure 3C). A strong positive correlation (*r* = 0.955) was observed among 38 of transcription-sync proteins (Figures 3D,E), but a weak positive correlation (*r* = 0.194) for 16 transcription-sync proteins (Figure 3F). Transcription-nonsync proteins showed a negative/inverse correlation (Figure 3G; *r* = 0.403).

### Disproportionate Transcription vs. Translation in the CaNrf2-TG Hearts Is Strongly Coupled With the RS Induced Cardiac Structural and Functional Dyssynchrony

**Figure 4:**
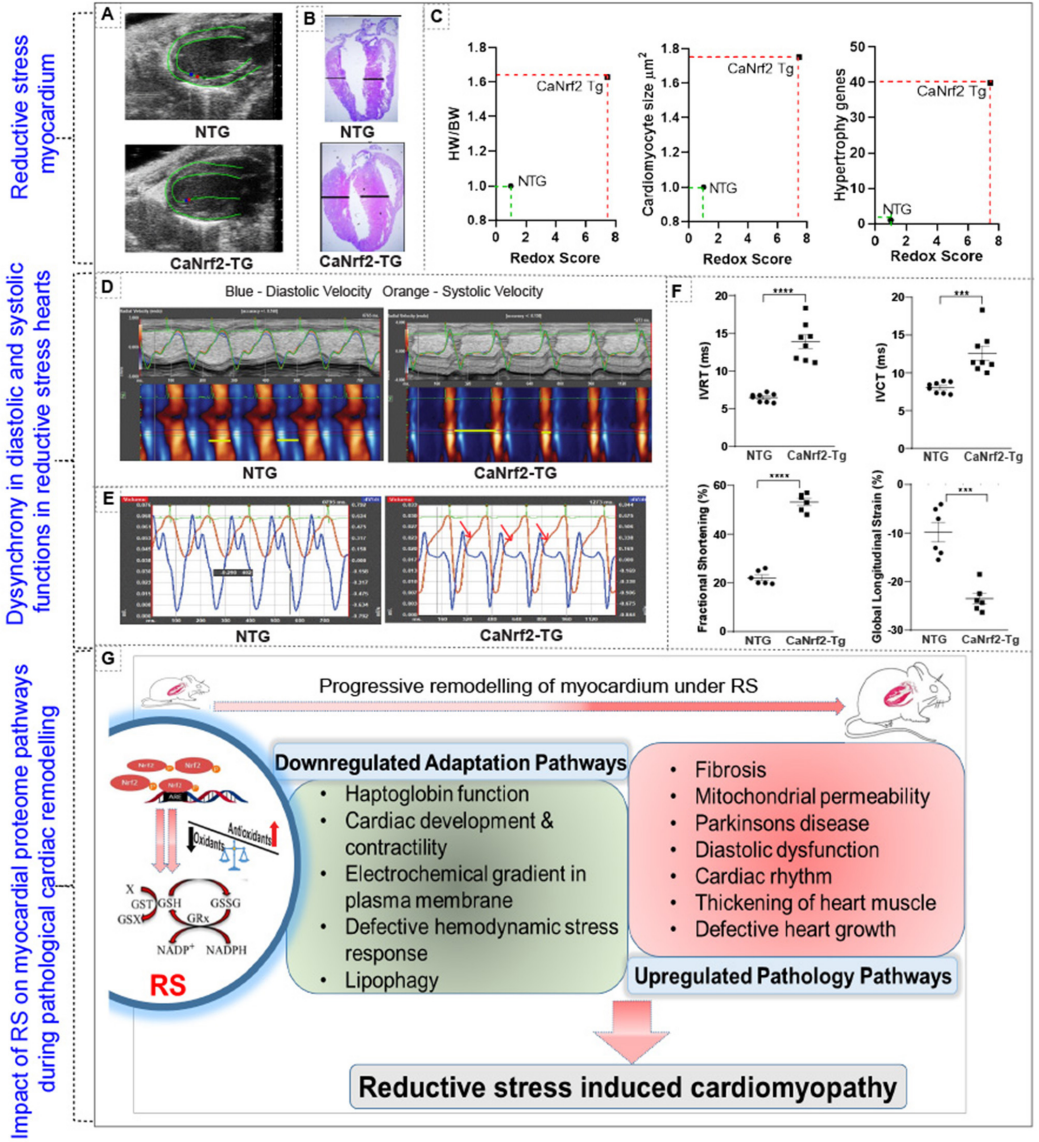
Impact of reductive stress on myocardial proteome signaling and pathological structural and functional remodeling. **(A)** Speckle tracking tracing showing deformation in the myocardial structure at longitudinal plane (PSLAX axis). **(B)** Morphology of hearts from autopsy of TG and NTg heart. **(C,D)** Correlating the impact of RS on structural remodeling at cellular (i.e. cardiomyocytes cell size), organ (i.e. heart/body weight ratio) and molecular (i.e. hypertrophy markers) level. **(D)** Cardiac function by speckle tracking echo analysis for wall motion- diastolic and systolic velocity, and **(E)** MVE and MVA waves and **(F)** other parameters fractional shortening, global longitudinal strain, isovolumic relaxation time (IVRT) and isovolumic contraction time (IVCT). **(G)** Schematic representation of altered proteome profiles/pathways and their impact on cardiac pathology during chronic RS. ***p < 0.001; ****p < 0.0001.

Our comparative analysis of RNA and protein in caNrf2-TG hearts demonstrated that 50% of the proteins do not match with the quantitative changes of their genes (mRNA) (Figure 3). Next, we explored whether these changes were associated with progressive structural and functional remodeling of the RS hearts. Speckle tracking tracing showed deformation in the myocardial structure at longitudinal plane (PSLAX axis) (Figure 4A; *n* = 6/gp.). Morphology of hearts from autopsy confirm the presence of hypertrophy in TG hearts (Figure 4B; *n* = 3/gp.). Consistent with the cRS associated structural remodeling in TG hearts, we noticed an increase in cardiomyocyte size, HW/BW ratio and gene-markers of hypertrophy (Figure 4C; *n* = 6–8/gp.). Analysis of cardiac function by novel STE echo demonstrated an impaired wall motion (diastolic and systolic velocity) pattern in TG hearts (Figure 4D; *n* = 6/gp.) with an elevated isovolumic relaxation/contraction time (IVRT/IVCT), which represents altered MV E/A waves (Figure 4E; *n* = 6/gp.). As we reported earlier using conventional echocardiography, the strain analysis reconfirmed that the TG hearts exhibited increased fractional shortening (20% vs. 50% in NTg vs. TG; *p* < 0.0001), decreased global longitudinal strain (−10% vs. −25%, *p* < 0.0002), increased IVRT (6.46 vs. 13.8 ms; *p* < 0.0001) and IVCT (8.06 vs. 12.5 ms; *p* < 0.0005) in comparison with NTg hearts (Figure 4F). Changes in the systolic and diastolic functions with structural abnormalities demonstrate cardiac dyssynchronicity in TG mice due to chronic RS. Of note, through TMT proteomics, we identified that while the adaptive signaling pathways were downregulated, the pathological signaling mechanisms were upregulated in the TG/RS hearts (Figure 4G).

## Discussion

Constitutive activation of Nrf2 under normal (unstressed) condition is a key inducer of RS (1, 22, 23). Our previous data on CaNrf2-TG mouse hearts with RS revealed a distinct transcriptome profile (1, 8). To elucidate the proteome of RS hearts, we employed TMT based mass spectrometric analysis and investigated i the differentially expressed proteins (DEPs). Proteomic profiling and computational pathway analyses provide both overall and specific information associated with RS pathology. Distinct segregation of a total of 1,105 proteins between the groups, as seen from PCA and cluster analysis, validates the RS dependent protein profile in caNrf2-TG mouse myocardium.

Gene ontology pathway analysis using Panther (24, 25) categorized oxidoreductase enzymes as the most enriched pathway along with pathways related to structural/functional adaptions during cardiac development. Prediction of direct (physical) and indirect (functional) association between proteins using String and Scaffold respectively (26) showed clustering and enrichment of NADPH associated proteins suggesting a possible development of reductive environment in TG hearts. Refining closely interacting protein families using K-means-based clustering showed three different clusters with enrichment of ubiquitin, GSR, NADPH family and stress proteins in the core proteome. Unbiased refining of the whole proteome showed high enrichment of metabolic enzymes crucial for redox homeostasis and proteins involved in redox signaling pathways. These results support distinct reductome (reductive stress proteome) signatures in CaNrf2-TG hearts. Among the DEPs responding to RS, several proteins were modified in the TG when compared to NTg hearts. Modifications like N-ethylmaleimide (NEM), oxidation, methylation and acetylation were obvious in TG/RS hearts. While modifications such as methionine loss, acetylation and cysteine oxidation are directly counted, NEM-adducts indicate the free thiol groups that are present only in the TG. As detailed in the methodology, NEM stably binds to free thiol groups and form NEM-adducts. There are 125 peptides bound with NEM in TG vs. NTg mice and these modifications indicate that these sites remained reduced (not oxidized in TG) *in vivo* due to reductive stress.

We also observed several upregulated proteins with no modifications, and down regulated proteins with or without modifications in CaNrf2 cardiac proteome; robust enrichment of glutathione and its related family proteins in TG hearts is noteworthy. The capacity to recycle GSH makes the glutathione system pivotal to the antioxidant defense mechanism and preserves cellular thiols (27). Proteome analysis revealed multiple modifications in more than one peptide of GSH family proteins (oxidation of cysteine in GSTA1 and GSTA2). While GSTA3 displays methionine oxidation, GSTA4 has lysine oxidation in one of the peptide fragments. However, in GSTM1, GSTM2 and GSTM3, both methionine and lysine residues were modified. Modifications in the cysteine residues of these GST family enzymes might impair their kinetics in the RS myocardium (28, 29). Noxious effects of such modifications in free cysteine residues that are in queue for tRNA selection may dramatically alter the translation process under RS (30, 31).

A comparison of RNA-seq data (NGS) with TMT proteomic data in CaNrf2 hearts indicated that an RS myocardium does not follow quantitative omics. Observed changes in the expression of proteins in response to RS might be primarily caused by (a) direct trans-activation of target genes of Nrf2, (b) chronic impact of RS, (c) RS-mediated posttranslational changes and (d) positive- or negative- feedback of protein synthesis rate on transcription of the respective gene(s). We hypothesize that changes in translational efficiency are caused, in part, by aggregation of proteins that might trigger a feedback inhibition of translation, or due to modified amino acids, which alter ribosomal attachment and decoding of mRNA resulting in synthesis of partial or overabundant transcript levels during translation (32–35). Moreover, modified peptides in the CaNrf2-TG may change the redox status through formation of mixed disulfide bonds, which then lead to irreversible protein aggregation in the myocardium (36). Of note, multiple steps between transcription and translation may provide different regulatory or pathological check points in these hearts, which needs further investigation (37, 38). Speckle-tracking based strain analysis of longitudinal global strain curves are altered in TG/RS hearts, which is a typical pattern seen in myocardial infarction or mechanical stress that leads to cardiac remodeling (39, 40).

For the first time, we report that a RS myocardium displays over 50% mismatch between mRNA and protein levels, warranting investigations using high-throughput transcriptome, proteomic and molecular approaches to mechanistically understand the impact of RS. This study points to new directions for future investigations on molecular signaling of RS and to identify novel diagnostic markers for RS cardiomyopathy. Thus, based on the findings between the association of redox milieu and altered structural/functional parameters (by STE) and morphometry, we highlight that chronic RS could be a novel mechanism for disproportionate transcription vs. translation, dysynchronous wall motion and altered systolic/diastolic functions in the TG hearts.

### Translational Impact

Nrf2 is crucial to maintain myocardial redox balance and proteome. We postulate that routine intake of over-the-counter antioxidants may recapitulate the caNrf2-TG mouse model developing RS. Under RS, we observed downregulation of several myocardial adaptation/rescue pathways and upregulation of pathophysiological processes, which are associated with RS cardiomyopathy over time. Thus, our results provide a rationale to develop personalized antioxidant therapeutic strategies to avoid RS mediated proteome alterations in humans.

### Limitations of the Study

Segmental STE analysis is warranted to identify the contractile abnormalities in relevant regions of the myocardium in response to RS.

## Data Availability Statement

The authors acknowledge that the data presented in this study must be deposited and made publicly available in an acceptable repository, prior to publication. Frontiers cannot accept a article that does not adhere to our open data policies.

## Ethics Statement

The animal study was reviewed and approved by University of Alabama IACUC #10160.

## Author Contributions

The study was designed by NR. TMT analysis was performed by CD and KP. STE echo analysis was performed by SS and AV. Methods for redox scoring developed by DJ and NR. SS, AV, and NR interpreted the data and wrote the manuscript. All authors read and approved the final version of this manuscript.

## Funding

This study was peripherally supported by funding from NHLBI (2HL118067 and HL118067) and NIA (AG042860) and the start-up funds (for NR) by the Department of Pathology and School of Medicine, the University of Alabama at Birmingham, AL, and UAB-AMC21 grant by the University of Alabama at Birmingham, AL and the Stony Wold-Herbert Fund (for AV).

## Conflict of Interest

The authors declare that the research was conducted in the absence of any commercial or financial relationships that could be construed as a potential conflict of interest.

## Publisher's Note

All claims expressed in this article are solely those of the authors and do not necessarily represent those of their affiliated organizations, or those of the publisher, the editors and the reviewers. Any product that may be evaluated in this article, or claim that may be made by its manufacturer, is not guaranteed or endorsed by the publisher.
