# Supplementary material for: Tandem Mass Tagging Based Identification of Proteome Signatures for Reductive Stress Cardiomyopathy
Source: Front Cardiovasc Med. 2022 Jun 13;9:848045. doi: 10.3389/fcvm.2022.848045 (PMC9234166; doi:10.3389/fcvm.2022.848045)
Supplement: Supplementary file 1 [file Data_Sheet_1.pdf]

Tandem Mass Tagging based identification of proteome signatures for reductive stress cardiomyopathy

Sini Sunny[a], Arun Jyothidasan[a], Cynthia L. David[b], Krishna Parsawar[b], Arul Veerappan[c,d], Dean P. Jones[e] , Steven Pogwizd[f] and Namakkal S. Rajasekaran[a,,g,h#]

[a]Molecular and Cellular Pathology, University of Alabama at Birmingham, Birmingham, AL, USA

[b]Analytical and Biological Mass Spectrometry Core Facility, University of Arizona, Tuscon, USA

[c]Division of Pulmonary, Critical Care and Sleep Medicine, Department of Medicine, New York University School of Medicine, New York, NY, USA.

[d]Department of Environmental Medicine, New York University School of Medicine, New York, NY, USA.

[e]Division of Pulmonary, Allergy, Critical Care and Sleep Medicine, Emory University, Atlanta, Georgia, USA.

[f] Comprehensive Cardiovascular Center, Department of Medicine, University of Alabama at Birmingham, Birmingham, AL, USA

[g]Division of Cardiovascular Medicine, Department of Medicine, University of Utah, Salt Lake City, UT, USA

[h]Center for Free Radical Biology, University of Alabama at Birmingham, Birmingham, AL, USA

[#]Correspondence:

Namakkal S. Rajasekaran, PhD

Center for Free Radical Biology

Division of Molecular & Cellular Pathology, Department of Pathology

The University of Alabama at Birmingham, BMR2 Room 533, 901 19th Street South, Birmingham, AL, 35294-2180, USA.

E-mail address: rajnsr@uabmc.edu (N.S. Rajasekaran)

Materials and Methods

Animals

Heart-specific constitutively active Nrf2 (CaNrf2) transgenic (TG) and non-transgenic (NTG) mice (n=4/group) at 6 months of age were used for analyzing the myocardial proteome. Animals were maintained under hygienic conditions with a 12 h day/night cycle, and fed with standard animal chow and water *ad libitum*. All studies were conducted in accordance with the Guide for Care and Use of Laboratory Animals developed by the National Research Council at the National Institutes of Health (NIH). The Institutional Animal Care and Use Committee (IACUC#14-10160) at the University of Alabama at Birmingham has approved the study.

Redox score calculation

A comprehensive approach was used to calculate the "redox score" in TG by comparing to basal redox factors in NTG (1-6). Here, we included the levels of (a) small molecular antioxidants (i.e. GSH, Cysteine/Cystine ratio); (b) antioxidant proteins (i.e. GSTμ, NQO1, CAT, GPX 1, SOD1, GCLM, GCLC, SOD2); (c) antioxidant enzymatic levels (ie. TAC) (d) antioxidant gene expression (ie. GCLM, NQO1, GSR, GST alpha, GCLC, GCLM, GSR, NQO1, GST MU, GPX 1, CAT) as well as the reactive oxygen species levels (ie. DHE). Next, we assessed the impact of RS on structural remodeling at (a) cellular level (ie. cardiomyocytes cell size); (b) organ level (ie. heart/body weight ratio) and (c) molecular level (ie. hypertrophy markers) by comparing the "redox score" with respective structural parameters (ie. RS vs CM size; RS vs HW/BW ratio; RS vs hypertrophy markers).

Sample preparation for Tandem Mass Tag (TMT) proteomic analysis

TMT-6plex reagents from Thermo were used to isotopically label peptides following reduction and alkylation of 60µg mouse heart lysate by following the vendor's protocols. Heart tissues were harvested from non-transgenic (NTG) & caNrf2-TG-High (TGH) (N=4 mice/group) after perfusing with N-ethylmaleimide (NEM; 10 µM). Heart tissue homogenates were prepared in RIPA buffer, and the cytosolic proteins were separated by centrifugation (5000 rpm, for 7 minutes at 4°C). Protein concentration was determined with a BCA kit (Bio-Rad, USA) according to the manufacturer's instructions. After trypsin digestion, peptides were desalted using a Strata-X C18 SPE column (Phenomenex, Torrance, CA, USA) and vacuum-dried. Peptides were then reconstituted in 0.5 M TEAB and processed according to the manufacturer's protocol using a TMT (Tandem Mass Tag) kit (ThermoFisher Scientific, Waltham, MA, USA). Briefly, one unit of TMT reagent was thawed and reconstituted in acetonitrile. The peptide mixtures were then incubated for 2 h at room temperature and pooled, desalted, and dried by vacuum centrifugation (7).

LC MS/MS Method and Data Searching

LC-MS/MS analysis was performed on a Q Exactive Plus mass spectrometer (Thermo Fisher Scientific, San Jose, CA) equipped with an EASY-Spray nanoESI source. Peptides (500ng) were eluted from an Acclaim Pepmap 100 trap column (75 micron ID x 2 cm, Thermo Scientific) onto an Acclaim PepMap RSLC analytical column (75 micron ID × 25 cm, Thermo Scientific) using a 3-25% gradient of solvent B (acetonitrile, 0.1% formic acid) over 150 min, 25-50% solvent B over 20 min, 50-70% of solvent B over 10 min, 70-95% B over 10 min then a hold of solvent 95% B for 20 min, and finally a return to 3% solvent B for 10 min. Solvent A consisted of water and 0.1% formic acid. Flow rates were

300 nL/min using a Dionex Ultimate 3000 RSLCnano System (Thermo Scientific). Data dependent scanning was performed by the Xcalibur v 4.0.27.19 software (8) using a survey scan at 70,000 resolution scanning mass/charge (m/z) 375-1400 at an automatic gain control (AGC) target of 3e6 and a maximum injection time (IT) of 50 msec, followed by higher-energy collisional dissociation (HCD) tandem mass spectrometry (MS/MS) at 32nce (normalized collision energy), of the 11 most intense ions at a resolution of 35,000, an isolation width of 1.2 m/z, an AGC of 1e5 and a maximum IT of 100 msec. Dynamic exclusion was set to place any selected m/z on an exclusion list for 30 seconds after a single MS/MS. Ions of charge state +1, 7, 8, >8, unassigned, and isotopes were excluded from MS/MS (9).

Protein identification

MS and MS/MS data were searched against the amino acid sequence of the Uniprot mouse protein database (10) and a common contaminant protein database (e.g., trypsin, keratins; obtained at ftp://ftp.thegpm.org/fasta/cRAP) using Thermo Proteome Discoverer v 2.4.0.305 (Thermo Fisher Scientific). MS/MS spectra matches considered fully tryptic peptides with up to 2 missed cleavage sites. Variable modifications considered were methionine oxidation (15.995 Da), and cysteine carbamidomethylation (57.021 Da). Proteins were identified at 95% confidence with XCorr score cut-offs (11) as determined by a reversed database search.

Quantitative data analysis

The protein and peptide identification results were further analyzed with Scaffold Q+S v 4.11.1 (Proteome Software Inc., Portland OR), a program that relies on results from search engines like Sequest, X!Tandem and MASCOT and which uses Bayesian

statistics to reliably identify more spectra (9). Protein identifications were accepted that passed a minimum of two peptides identified at 95% protein and peptide confidence levels. Quantitative values were generating using the Proteome Scaffold Q module. Acquired intensities in the experiment were globally normalized across all acquisition runs. Individual quantitative samples were normalized within each acquisition run. All acquisitions include n=4 biological samples in each group. Each individual sample was labelled using a TMT reagent with specific mass (i.e. 126, 127, 128, 129), then the NTG and TG samples were pooled for the run. Two independent runs were performed through tandem mass spectrometry, and the data from two different runs was averaged for the given experimental group. An intensity-based normalization of MS samples was carried out. Intensities for each peptide identification were normalized within the assigned protein. The reference channels were normalized to produce a 1:1 fold change. The data, analytic methods, and study materials will be made available to researchers for purposes of reproducing the results or replicating the procedure. The mass spectrometry proteomics data have been deposited to the ProteomeXchange Consortium (http:// proteomecentral.proteome xchange.org) via the PRIDE partner repository.

Next generation RNA sequencing

Total RNA was isolated from TG and NTG mice (n=4/group) hearts at 6 months of age using the RNeasy Mini Kit (Qiagen, Cat.74106) according to the manufacturer's instructions. The purity of RNA was confirmed using Bio-analyzer and intact poly(A) transcripts were purified from total RNA using oligo(dT) magnetic beads and mRNA sequencing libraries were prepared with the TruSeq Stranded mRNA Library Preparation Kit (Illumina, RS-122-2101, RS122-2102) (12, 13).

Immunoblotting

Independent validation of some of the MS data was carried out using protein specific antibodies. Heart tissues from NTG and caNrf2 TG mice (n = 4/group) at 6 months of age were homogenized in a cytosolic extraction buffer (10 mM HEPES, 10 mM KCl, 0.1 mM EDTA, 0.5 mM $MgCl_2$, with freshly prepared 0.1 mM phenyl methylsulfonyl fluoride (PMSF), 1 mM dithiothreitol, and 1% Triton X-100, pH 7.9) and centrifuged at 5000 rpm for 5–6 min. Protein concentrations were determined with the Bradford reagent, and equal amounts of protein were resolved on 10%–12% sodium dodecyl sulfate–polyacrylamide gel electrophoresis. Proteins were then transferred to polyvinylidene difluoride membranes (EMD Millipore Corp., Billerica, MA) and blocked-in tris-buffered saline-Tween 20 (TBST) containing 5%–10% nonfat dry milk or BSA. Membranes were then incubated overnight at 4°C with their respective primary antibodies (1%–2% BSA in TBST) followed by horseradish peroxidase IgG (Vector Laboratories)-conjugated secondary antibody (anti-rabbit and anti-mouse, 0.2% BSA in TBST) incubation followed by chemiluminescence-based detection (Pierce, Rockford, IL) with an Amersham Imager 600 (GE Healthcare Life Sciences, Chicago, IL) (6, 14).

Bioinformatics tools

Differentially expressed proteins (DEPs) were then plotted for heat maps based on fold change with R studio package "pheatmap" (Version 1.2.5033). PCA plot was plotted using ClustVis (https://biit.cs.ut.ee/clustvis/). We performed Protein Analysis THrough Evolutionary Relationships (PANTHER) (pantherdb.org) to predict the functions of uncharacterized proteins in TMT proteome, based on their evolutionary relationships. Also, we carried out STRING analysis (https://string-db.org/), to predict protein-protein

interactions - direct (physical) and indirect (functional) associations between the proteins analyzed in the scaffold software (13).

Speckle tracking echocardiography (STE)

Echocardiography was performed on both adult NTG and TG mice at 6 months of age (n=8) under light sedation (1-2% isoflurane in oxygen) using a 38 MHz linear-array transducer with a digital high-resolution ultrasound system (Vevo 2100 Imaging System, Visual Sonics, Toronto, Canada) (1). Conventional and novel speckle tracking based strain echocardiographic image measurements were performed offline. All image acquisitions and offline measurements included in the present STE analysis were conducted by a single investigator who was blinded to animal groups. Echocardiographic images were acquired at the highest frame rate possible (232 frames/s) and strain analysis was performed in the longitudinal axes (15-17). Three consecutive cardiac cycles were selected for analysis, and semi automated tracing of the endocardial and epicardial borders were performed and verified over all 3 cardiac cycles in each cine loop. Strain measures were averaged over the obtained cardiac cycles, resulting in curvilinear strain (Figure 4F). Images obtained from Vevo Strain software were used to represent the systolic/diastolic velocity change and MVE/MVA pattern change in caNrf2 Tg vs NTG. After the echocardiography, mice were euthanized, and hearts were extracted for protein and RNA isolation as well as histopathological assessment of cardiac remodeling.

Statistics

Proteomic data: p values (<0.05) for differentially expressed proteins were calculated using Bonferroni Corrected Mann–Whitney tests (Scaffold 4.0). *Western blotting*: Mean ± standard error is shown (n = 4 hearts per experimental group). Comparisons were

performed by one-way ANOVA. $P < 0.05$ was considered statistically significant.

Abbreviations Used

BIN1 – Bridging Integrator 1; BLVRB – Biliverdin reductase B; CaNrf2-Tg Constitutively active Nrf2 transgenic; cRS Chronic reductive stress; DEPs Differentially expressed proteins; FDR False Discovery Rate; GPX Glutathione peroxidase; GSR Glutathione reductase; GSTA Glutathione S-Transferase Alpha; GSTM Glutathione S-Transferase Mu; HW/BW Heart weight/body weight; IVRT/ IVCT Isovolumic relaxation/contraction time; NADPH Reduced nicotinamide adenine dinucleotide phosphate; NEM N-ethylmaleimide; NQO1 NADPH Quinone Oxidoreductase 1; Nrf2 Nuclear factor erythroid 2 like 2; NTg Non-transgenic; PIR Pirin; PRDX6 Peroxiredoxin 6; PSLAX Parasternal Long Axis; ROS/RNS Reactive oxygen and nitrogen species; RS Reductive Stress; SOD Superoxide Dismutase; STE Speckle tracking echocardiography; TALDO1 Transaldolase 1; TMT Tandem mass tagging; T-SP Transcription-sync proteins

References

1. Shanmugam G, Wang D, Gounder SS, Fernandes J, Litovsky SH, Whitehead K, et al. Reductive Stress Causes Pathological Cardiac Remodeling and Diastolic Dysfunction. Antioxid Redox Signal. 2020;32(18):1293-312.
2. Shanmugam G, Challa AK, Devarajan A, Athmanathan B, Litovsky SH, Krishnamurthy P, et al. Exercise Mediated Nrf2 Signaling Protects the Myocardium From Isoproterenol-Induced Pathological Remodeling. Front Cardiovasc Med. 2019;6:68.
3. Shanmugam G, Challa AK, Litovsky SH, Devarajan A, Wang D, Jones DP, et al. Enhanced Keap1-Nrf2 signaling protects the myocardium from isoproterenol-induced pathological remodeling in mice. Redox Biol. 2019;27:101212-.
4. Quiles JM, Narasimhan M, Mosbruger T, Shanmugam G, Crossman D, Rajasekaran NS. Identification of transcriptome signature for myocardial reductive stress. Redox Biol. 2017;13:568-80.
5. Kannan S, Muthusamy VR, Whitehead KJ, Wang L, Gomes AV, Litwin SE, et al. Nrf2 deficiency prevents reductive stress-induced hypertrophic cardiomyopathy. Cardiovasc Res. 2013;100(1):63-73.
6. Shanmugam G, Narasimhan M, Tamowski S, Darley-Usmar V, Rajasekaran NS. Constitutive activation of Nrf2 induces a stable reductive state in the mouse myocardium. Redox Biol. 2017;12:937-45.

7. Andon NL, Hollingworth S, Koller A, Greenland AJ, Yates JR, 3rd, Haynes PA. Proteomic characterization of wheat amyloplasts using identification of proteins by tandem mass spectrometry. Proteomics. 2002;2(9):1156-68.
8. Andon NL, Hollingworth S, Koller A, Greenland AJ, Yates III JR, Haynes PA. Proteomic characterization of wheat amyloplasts using identification of proteins by tandem mass spectrometry. PROTEOMICS: International Edition. 2002;2(9):1156-68.
9. Keller A, Nesvizhskii AI, Kolker E, Aebersold R. Empirical statistical model to estimate the accuracy of peptide identifications made by MS/MS and database search. Anal Chem. 2002;74(20):5383-92.
10. Cottrell JS. Protein identification using MS/MS data. Journal of Proteomics. 2011;74(10):1842-51.
11. Downs CA, Johnson NM, Tsaprailis G, Helms MN. RAGE-induced changes in the proteome of alveolar epithelial cells. J Proteomics. 2018;177:11-20.
12. Quiles JM, Narasimhan M, Mosbruger T, Shanmugam G, Crossman D, Rajasekaran NS. Identification of transcriptome signature for myocardial reductive stress. Redox Biol. 2017;13:568-80.
13. Sunny S, Challa AK, Qiao A, Jyothidasan A, Krishnamurthy P, Ramamurthy MT, et al. Transcriptional Regulation of Structural and Functional Adaptations in a Developing Adulthood Myocardium. Cardiol Cardiovasc Med. 2021;5(5):454-70.
14. Shanmugam G, Challa AK, Litovsky SH, Devarajan A, Wang D, Jones DP, et al. Enhanced Keap1-Nrf2 signaling protects the myocardium from isoproterenol-induced pathological remodeling in mice. Redox Biol. 2019;27:101212.
15. Walsh-Wilkinson E, Drolet MC, Arsenault M, Couet J. Sex differences in the evolution of left ventricle remodeling in rats with severe volume overload. BMC Cardiovasc Disord. 2020;20(1):51.
16. Dandel M, Lehmkuhl H, Knosalla C, Suramelashvili N, Hetzer R. Strain and strain rate imaging by echocardiography - basic concepts and clinical applicability. Curr Cardiol Rev. 2009;5(2):133-48.
17. Smiseth OA, Torp H, Opdahl A, Haugaa KH, Urheim S. Myocardial strain imaging: how useful is it in clinical decision making? Eur Heart J. 2016;37(15):1196-207.

Figure legends

Supplemental Figure 1: (A) TMT spectrum for 1105 proteins**.** (B) Details for scaffold software, databases and criteria for protein identification.

Supplemental Figure 2: Gene ontology pathway by STRING analysis. STRING analysis (https://string-db.org/) predicted the protein-protein interactions – showing different subsets of interaction(s) with ubiquitin, GSR, stress proteins, NADPH as the top

enriched networks in TG heart. The network has been refined using K means clustering method with a highest confidence cut off 0.900. Proteins' nodes are colored automatically based on the number of clusters opted.
