# Supplementary material for: Tandem Mass Tagging Based Identification of Proteome Signatures for Reductive Stress Cardiomyopathy
Source: Front Cardiovasc Med. 2022 Jun 13;9:848045. doi: 10.3389/fcvm.2022.848045 (PMC9234166; doi:10.3389/fcvm.2022.848045)
Supplement: Supplementary file 2 [file Image_1.pdf]

Supplemental Figure 1

A

NSR_3788660_TMT_1_6_240min 01/27/21 18:11:58
500ng

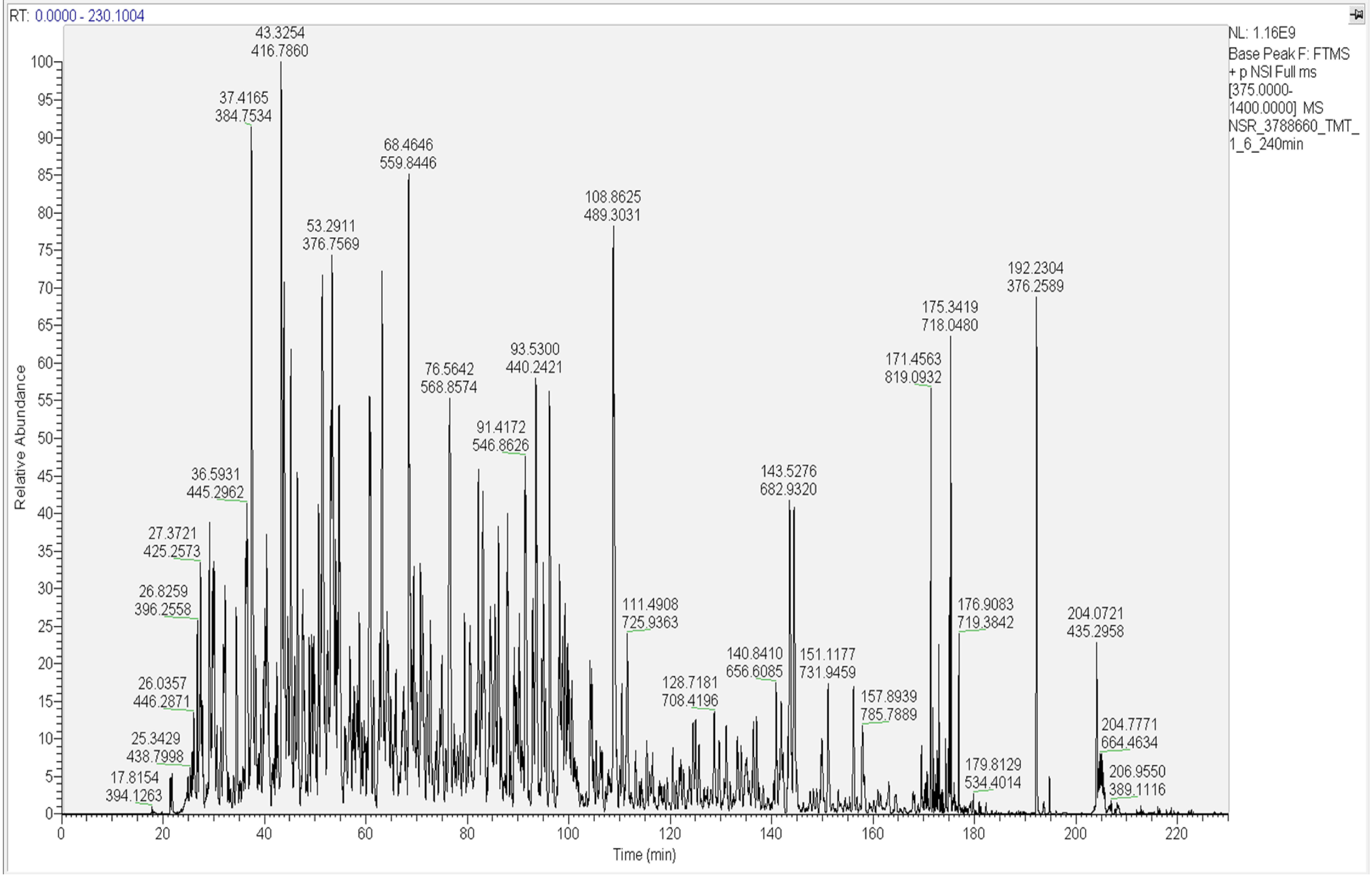

B

Experiment Methods

Parameter	Value
Experiment:	NSR_3788660 (1)
Peak List Generator:	
Version:	
Charge States Calculated:	
Deisotoped:	
Textual Annotation:	
Database Set:	1 Database
Database Name:	Contaminants.fasta; Mus_Musculus_Uniprot_Rev_17042_07292020.fasta
Version:	
Taxonomy:	All Entries
Number of Proteins:	17263
Does database contain common contaminants?:	
Search Engine Set:	1 Search Engine
Search Engine:	Sequest
Version:	IseNode in Proteome Discoverer 2.4.0.305
Samples:	All Samples
Fragment Tolerance:	0.020 Da (Monoisotopic)
Parent Tolerance:	10.0 PPM (Monoisotopic)
Fixed Modifications:	+229 on K (TMT6plex)
Variable Modifications:	-131 on M (Met-loss), -89 on M (Met-loss+Acetyl), +16 on M (Oxidation), +...
Database:	Contaminants.fasta; Mus_Musculus_Uniprot_Rev_17042_07292020.fasta (...
Digestion Enzyme:	Trypsin
Max Missed Cleavages:	2
Probability Model:	
NSR_3788660_TMT_1_6_240min_Static:	Use Previously Calculated Probability (No analysis) [all charge states]
NSR_3788660_TMT_7_12_240min_Static:	Use Previously Calculated Probability (No analysis) [all charge states]
Scaffold:	Version: Scaffold_4.11.1
Modification Metadata Set:	2334 modifications
Source:	C:\Program Files\Scaffold 4\parameters\unimod.xml
Comment:	
Protein Grouping Strategy:	Experiment-wide grouping with protein cluster analysis
Peptide Thresholds:	95.0% minimum
Protein Thresholds:	95.0% minimum and 2 peptides minimum
Peptide FDR:	0.0% (Decoy)
Protein FDR:	0.1% (Decoy)
GO Annotation Source(s):	
Pathway Annotation Source(s):	
Alternate ID Source(s):	FASTA:UniProt/Swiss-Prot Alt. Accession (UniProtKB)

DATABASE SEARCHING-- Tandem mass spectra were extracted by [unknown] version [unknown]. Charge state deconvolution and deisotoping were not performed. All MS/MS samples were analyzed using Sequest (Thermo Fisher Scientific, San Jose, CA, USA; version IseNode in Proteome Discoverer 2.4.0.305). Sequest was set up to search Contaminants.fasta; Mus_Musculus_Uniprot_Rev_17042_07292020.fasta (unknown version, 17263 entries) assuming the digestion enzyme trypsin. Sequest was searched with a fragment ion mass tolerance of 0.020 Da and a parent ion tolerance of 10.0 PPM. TMT6plex of lysine was specified in Sequest as a fixed modification. Met-loss of methionine, met-loss+Acetyl of methionine, oxidation of methionine, acetyl of the n-terminus and nethylmaleimide of cysteine were specified in Sequest as variable modifications.

CRITERIA FOR PROTEIN IDENTIFICATION-- Scaffold (version Scaffold_4.11.1, Proteome Software Inc., Portland, OR) was used to validate MS/MS based peptide and protein identifications. Peptide identifications were accepted if they could be established at greater than 95.0% probability by the Percolator posterior error probability calculation (Käll, L et al, Bioinformatics, 24(16):i42-i48, Aug 2008). Protein identifications were accepted if they could be established at greater than 95.0% probability and contained at least 2 identified peptides. Protein probabilities were assigned by the Protein Prophet algorithm (Nesvizhskii, AI et al Anal. Chem. 2003;75(17):4646-58). Proteins that contained similar peptides and could not be differentiated based on MS/MS analysis alone were grouped to satisfy the principles of parsimony. Proteins sharing significant peptide evidence were grouped into clusters.
