# Supplementary figures and images for: Tandem Mass Tagging Based Identification of Proteome Signatures for Reductive Stress Cardiomyopathy
Source: Front Cardiovasc Med. 2022 Jun 13;9:848045. doi: 10.3389/fcvm.2022.848045 (PMC9234166; doi:10.3389/fcvm.2022.848045)

Supplemental Figure 2

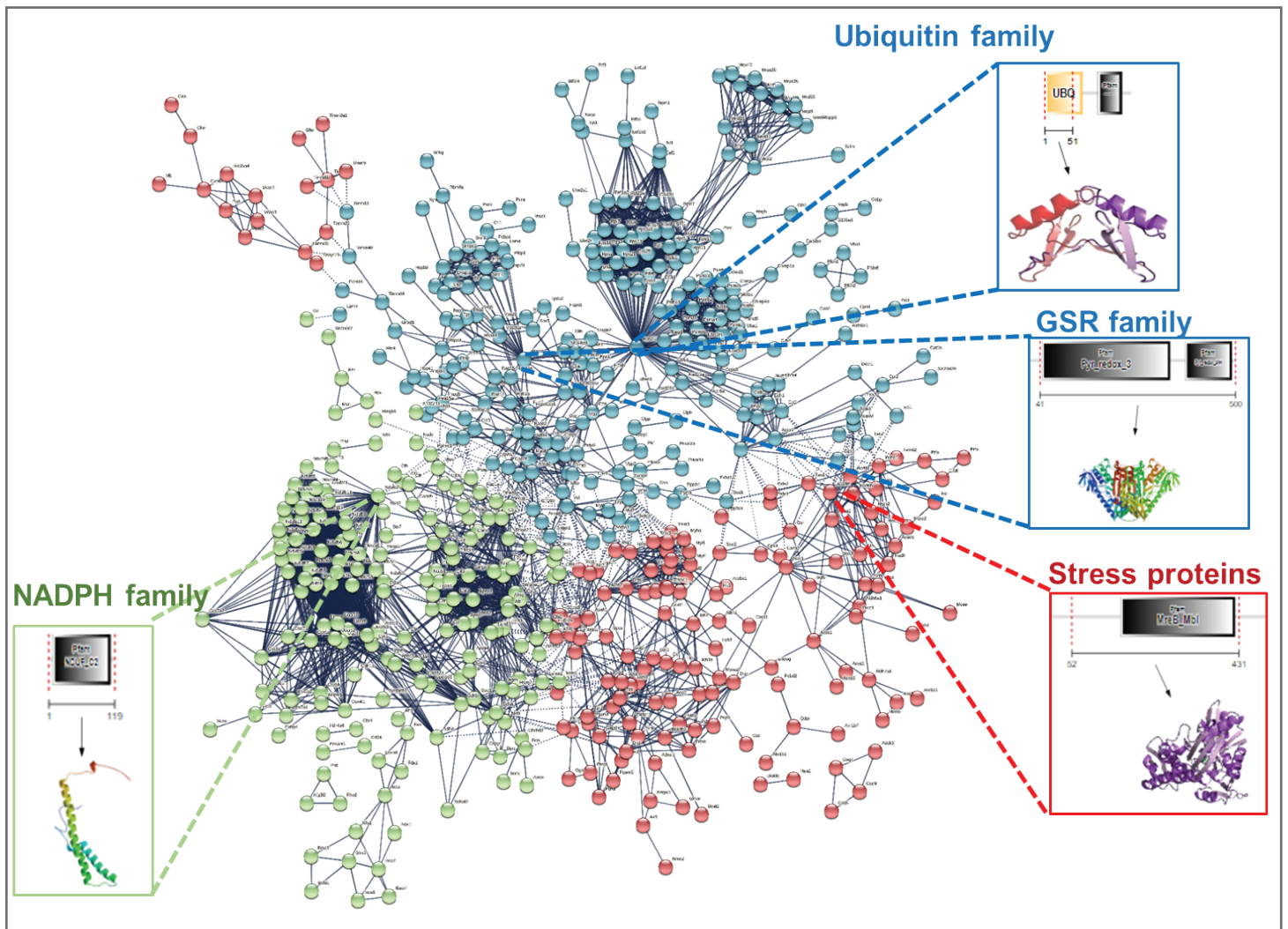

Supplement: Supplementary file 3 [file Image_2.pdf]
